# The sick building syndrome

**DOI:** 10.4103/0019-5278.43262

**Published:** 2008-08

**Authors:** Sumedha M. Joshi

**Affiliations:** Department of Preventive and Social Medicine, Dr D. Y. Patil Medical College, Nerul, Navi Mumbai, India

**Keywords:** Building, sick, syndrome

## Abstract

The sick building syndrome comprises of various nonspecific symptoms that occur in the occupants of a building. This feeling of ill health increases sickness absenteeism and causes a decrease in productivity of the workers. As this syndrome is increasingly becoming a major occupational hazard, the cause, management and prevention of this condition have been discussed in this article.

## INTRODUCTION

The sick building syndrome (SBS) is used to describe a situation in which the occupants of a building experience acute health- or comfort-related effects that seem to be linked directly to the time spent in the building. No specific illness or cause can be identified. The complainants may be localized in a particular room or zone or may be widespread throughout the building.[1]

## CLASSIFICATION

### The health conditions associated with buildings are commonly classified as:

1. SBS or Tight building syndrome.
2. Building-related disease, when the symptoms of diagnosable illness are identified and attributed directly to airborne building contaminants.
3. Building-associated symptoms.[2]

### Signs and symptoms of the sick building syndrome are as follows[3]

Headache, dizziness, nausea, eye, nose or throat irritation, dry cough, dry or itching skin, difficulty in concentration, fatigue, sensitivity to odours, hoarseness of voice, allergies, cold, flu-like symptoms, increased incidence of asthma attacks and personality changes.

The cause of the symptoms is not known. It reduces work efficiency and increases absenteeism. Most of the complainants report relief soon after leaving the building, although lingering effects of neurotoxins can occur.

### Signs and symptoms of the building-related disease are as follows

Cough, chest pain, shortness of breath on mild exertion, edema, palpitations, nosebleeds, cancers, pregnancy problems and miscarriages. Extrinsic allergic alveolitis, Legionnaire's disease, humidifier fever, pneumonia and occupational asthma are also known to occur.

Legionnaire's disease is due to contamination of cooling towers by legionella organisms. Legionella is also responsible for Pontiac fever. Legionnaire's disease occurs predominantly in the middle aged and elderly adults whereas Pontiac fever occurs in young healthy adults, and has a very high secondary attack rate.[4]

Humidifier fever is caused by breathing in water droplets from humidifiers heavily contaminated with microorganisms causing respiratory infections, asthma and extrinsic allergic alveolitis. The disease is noninfective in nature. The patient may have flu-like symptoms. It is sometimes called Monday Fever. Permanent lung damage does not occur.

The symptoms can be clinically defined and have clearly identifiable causes. The complainants may require prolonged recovery time after leaving the building.

It is important to note that complaints may also result from other cause like a preexisting illness or other allergies, job-related stress or dissatisfaction and psychosocial factors.[5]

## ETIOLOGY

The following are some of the factors that might be primarily responsible for SBS:

### 1. Chemical contaminants

1.1. From outdoor sources: Contaminants from outside like pollutants from motor vehicle exhaust, plumbing vents and building exhausts (bathrooms and kitchens) can enter the building through poorly located air intake vents, windows and other openings. Combustion byproducts can enter a building from a nearby garage. Radon, formaldehyde, asbestos, dust and lead paint can enter through poorly located air intake vents and other openings.
1.2. From indoor sources: The most common contaminant of indoor air includes the volatile organic compounds (VOC). The main sources of VOC are adhesives, upholstery, carpeting, copy machines, manufactured wood products, pesticides, cleaning agents, etc. Environmental tobacco smoke, respirable particulate matter, combustion byproducts from stove, fireplace and unvented space heater also increase the chemical contamination. Synthetic fragrances in personal care products or in cleaning and maintenance products also contribute to the contamination.

### 2. Biological contaminants

The biological contaminants include pollen, bacteria, viruses, fungus, molds, etc. These contaminants can breed in stagnant water that has accumulated in humidifiers, drainpipes and ducts or where water has collected on ceiling tiles, insulation, carpets and upholstery.

Insect and bird droppings can also be a source of biological contamination. Biological contamination causes fever, chills, cough, chest tightness, muscle aches and allergic reactions. In offices with a high density of occupancy, airborne diseases can spread rapidly from one worker to another. Air-conditioning systems can recirculate pathogens and spread them throughout the building e.g., Legionnaire's disease due to legionella organisms.[4,6]

### 3. Inadequate ventilation

In 1970, oil embargo led building designers to make buildings more airtight, with less outdoor air ventilation, in order to improve energy efficiency.[7] The ventilation was reduced to 5 cfm/person. This reduced ventilation rate was found to be inadequate to maintain the health and comfort of building occupants. Malfunctioning heating, ventilation and air-conditioning systems (HVAC systems) also increase the indoor air pollution. In order to have an acceptable indoor air quality (IAQ) with a minimum energy consumption, The American Society of Heating, Refrigeration and Air-Conditioning Engineers (ASHRAE) recently revised ventilation standards to a minimum outdoor air flow rate of 15 cfm/person to avoid the problems related to inadequate ventilation. The standards are 20 cfm/person in office spaces and 60 cfm/person in smoking lounges.[1] Poor design and construction of buildings with more number of offices cramped in a building to increase the salable area also contribute to inadequate ventilation.

### 4. Electromagnetic radiation

Gadgets like microwaves, televisions and computers emit electromagnetic radiation, which ionizes the air. Extensive wiring without proper grounding also creates high magnetic fields, which have been linked to cancer.

### 5. Psychological factors

Excessive work stress or dissatisfaction, poor interpersonal relationships and poor communication are often seen to be associated with SBS.

### 6. Poor and inappropriate lighting with absence of sunlight, bad acoustics, poor ergonomics and humidity may also contribute to SBS.

The symptoms of SBS are commonly seen in people with clerical jobs than in people with managerial jobs because professionals or managers have better working conditions. The symptoms are more common in females than in males probably because more females are in secretarial jobs, they are more aware of their health or a lesser dose of pollutants is required to manifest the effects. The symptoms are more common in air-conditioned buildings than in naturally ventilated buildings and are more common in a public sector building than in a private sector building [Table 1].[8]

**Table 1:** Types of problems found in 203 indoor air quality investigations carried out by National Institute for Occupational Safety and Hygiene

Problem	No.	%	Notes
Contamination (inside)	36	18	Exposure to chemical or other toxic agents generated within the office space, e.g., methyl alcohol from a spirit duplicator, methacrylate from a copier, sulfur dioxide from a heating system, amines used in a humidification system, chlordane used as a pesticide
Contamination (outside)	21	10	Exposure to a chemical or other toxic substance originating from a source outside the building, e.g., motor vehicle exhaust fumes, construction activity, underground petrol spillage
Contamination (building fabric)	7	3	Problems from the material used to construct the building (figure excludes asbestos), e.g., formaldehyde, fiberglass
Inadequate ventilation	98	48	Symptoms may be due to low levels of multiple contaminants and/or poor ventilation
Hypersensitivity pneumonitis	6	3	Problems due to a reaction to microorganisms in the building environment
Cigarette smoking	4	2	
Humidity	0.9	4	
Noise/illumination	2	1	
Scabies	1	0.5	
Unknown	19	9	

Source: Melius 1984

## INVESTIGATIONS

1. Evaluating the IAQ and identifying the contaminant by air sampling.
2. Establishing a cause and effect relationship between symptoms and IAQ.
3. Identifying the cause of the complaints so that appropriate corrective measures can be initiated.
4. Conducting a ‘walk-through’ inspection of the problems areas and collecting information on the following:
  4.1. the Occupants,
  4.2. HVAC Systems for pollution pathways and
  4.3. Possible contamination sources.[10]

The occupational health and safety resource centre at Canada's University of Western Ontario has devised a routine five-point survey for occupational hygienists to follow when investigating air quality complaints (Ruhemann 1985). Features include:

1. a walk-through inspection to look for sources of contamination, such as photocopiers, insulation and cleaning materials,
2. measurement of temperature, humidity, air movement and other comfort parameters,
3. measurement of carbon dioxide to assess the ventilation efficiency,
4. measurement of formaldehyde, carbon monoxide, ozone and respirable particles and
5. examination of the ventilation system for causes of poor distribution, including tests for biological organisms in any water in the system[9]

## PREVENTION AND CONTROL

1. Increase the ventilation rates and air distribution. The heating, ventilation and air-conditioning systems should be designed to meet ventilation standards in the local building codes. The HVAC system should be operated and maintained properly to ensure that the desired ventilation rates are attained. If there are strong pollutants, the air may need to be directly vented to the outside. This method is especially recommended to remove pollutants that accumulate in specific areas such as rest rooms, copy rooms and printing facilities. The ASHRAE recommends a minimum of 8.4 air exchanges per 24 h.
2. Removal or modification of the pollutant source can be carried out by a routine maintenance of HVAC systems, replacing water-stained ceiling tiles and carpets, using stone, ceramic or hardwood flooring, proper water proofing, avoiding synthetic or treated upholstery fabrics, minimizing the use of electronic items and unplugging idle devices, venting contaminants to the outside, storing paints, solvents, pesticides and adhesives in close containers in well-ventilated areas and using these pollutant sources in periods of low or no occupancy. Allowing time for building material in new areas to off-gas pollutants before occupancy and smoking restrictions are some measures that can be used.
3. Air cleaning can be a useful addition to control air pollution. Air cleaning can be performed by ensuring uncongested interiors with open office designs, use of frosted glass and skylights that give access to natural light, terrace gardens, community spaces and indoor plants that absorb carbon monoxide and formaldehyde from the air. Air filters are also effective in removing some if not all of the pollutants.
4. Education and communication are important parts of any air quality management programme so as to work more effectively and efficiently to prevent and solve the health problems.
5. Legislation
  Banning of smoking in the workplace or restricting smoking to designated well-ventilated areas away from the work stations and creating no-smoking zones with the help of laws. In some European countries, workers have a statutory right to be involved with the employer's plans for changes in the work place.
6. Research
  A field of study originating in Germany called Bau-biologie or Building biology has been initiated. The principles of Building biology are as follows:[11]
  6.1. Site status
    The building site should be geologically undisturbed. Residential areas should be away from industrial centers and main traffic routes and housing should have sufficient green space and should be in harmony with the surrounding environment.
  6.2. Construction concepts
    Natural, unadulterated and nontoxic building material should be used, walls, floors and ceilings should not be susceptible to mold or fungi, the basement should be waterproof and well -ventilated, the earth's natural magnetic field should not be altered or distorted, production, installation and disposal of building materials should not lead to environmental pollution, building activities should not lead to exploitation of nonrenewable, rate resources.
  6.3. Interiors
    Lighting and color must mix well with the surroundings and not jar the senses, man-made electromagnetic radiation must be reduced as much as possible, interiors should be done by using natural materials without toxic content and should be economically designed, there should be no toxic outgases or harsh smells, indoor humidity should be naturally regulated, air pollutants should be filtered and neutralized, thermal insulation should be balanced with heat retention, use of solar heating should be encouraged, moisture content in new buildings should be low, protective measures against noise pollution and harmful infrasonic and ultrasound radiation must be ensured, natural balance of atmospheric electricity and ion concentration should be maintained [Table 2].

**Table 2:** Comfort levels recommended by the Chartered Institution of Building Services Engineers (CIBSE 1986)

Parameter	Recommended level
Temperature (dry bulb)	19-23°C
Relative humidity (RH)	40-70%
	More than 55% RH is needed in carpeted buildings with under floor heating to avoid electrostatic shocks
*Ventilation*	
Delivery of fresh air	8 l/s/person (minimum)
	16 l/s/person where some smoking
	25 l/s/person where heavy smoking
Total air supply	4–6 air changes/h
Air speed	0.1–0.3 m/s. Less than 0.1 m/s causes stuffiness. More than 0.3 m/s causes draughts. For air speeds higher than 0.1 m/s, CIBSE recommends an increase in air temperature to take account of air movement
Sound	46 dBA is the upper limit for general office work
Lighting	500 lux for general office work, 750 lux for deep-plan offices and work at drawing boards, proofreading, etc.

## References

[1] Indoor Air Facts No. 4 (revised) Sick building syndrome. http://www.epa.gov/iaq/pubs/sbs.html.

[2] Purushottam K (2001). The sick building syndrome. Indian J Occup Health.

[3] Martin-Gil Outcome of research into a sick hospital. Hospital Management International.

[4] Breiman RF, Fraser DW (1992). Legionelloisis, Maxy-Rosenau – Last: Public Health and Preventive Medicine.

[5] Redlich CA, Sparer J, Cullen MR (1997). Sick building syndrome. Lancet.

[6] Phoon WO (1988). Practical occupational health.

[7] IAQ fact sheet sick building syndrome, Environmental Health Center. http://www.nsc.org/ehc/indoor/sbs.htm-8k-.

[8] Finnegan MJ, Pickering CA, Burge PS (1984). The sick building syndrome: Prevalence studies. Br Med J.

[9] Sick building syndrome: Causes, effects and control.

[10] IAQ publications Sick building syndrome fact sheet indoor air facts no. 4 (Revised). http://www.epa.gov/iaq/pubs/sub.html-29k-.

[11] (2004). ‘Room in the skies’ Outlook – life wellness.

